# A meta-analysis of semiconductor materials fabricated in microgravity

**DOI:** 10.1038/s41526-024-00410-7

**Published:** 2024-06-26

**Authors:** Ashley R. Wilkinson, Frances Brewer, Hannah Wright, Ben Whiteside, Amari Williams, Lynn Harper, Anne M. Wilson

**Affiliations:** 1 Department of Chemistry and Biochemistry, Butler University, Indianapolis, IN USA; 2 Ames Research Center, National Aeronautics and Space Administration, Moffett Field, CA USA

**Keywords:** Electronic devices, Materials for optics

## Abstract

This meta-analysis of 160 semiconductor crystals that were grown in microgravity on orbital vehicles between 1973 and 2016 is based on publicly available information documented in the literature. This analysis provides comparisons of crystal metrics including size, structure quality, uniformity, and improved performance between crystals grown in microgravity or terrestrially. Improvement in at least one of these metrics was observed for 86% of those materials that included data in their studies.

## Introduction

Microgravity research on fabrication of specialized materials has been a part of low earth orbit research since 1973^1^. Semiconductor crystals grown in microgravity show improved properties visually, grow larger, display structural improvements, have a more uniform composition among multiple crystals, and/or display superior performance over those grown terrestrially^2–5^. Exotic semiconductor production in space has the potential to benefit industry through optic, thermoelectric, infrared, and telecommunication applications^6^. Information about semiconductors fabricated in microgravity was performed in the late 1980s by Regel^1^, but no updated reports have been made. A larger dataset of inorganic compounds included semiconductors^5^. This current meta-analysis of semiconductor experimentation in microgravity is based on credible publicly available information documented in the literature between 1973 and 2016. The pertinent metrics found in these sources have been analyzed, including semiconductor material, flight, author(s), journal title, DOI, mission flown, year flown, country of PI, crystal shape, number of crystals, molecular weight, crystal size, unit cell parameters, space group, resistivity, methods, conditions, data reported, flight complications, materials, temperature of experiment, as well as additional relevant comments reported by the authors. The compiled data was evaluated utilizing crystal metrics including crystal size, structure quality, and uniformity, and improved performance between crystals grown in microgravity or terrestrially. Data as of July 22, 2023 is presented herein.

## Results

At the time of analysis, 160 semiconductor crystallization experiments in microgravity were performed; 89% (143) came from peer-reviewed journals. The remaining reports derive from graduate theses, NASA technical reports, symposia, and books, and an additional three have been independently verified by a subject matter expert, Dr. Jessica Frick, Stanford University. The compiled research represents 13 countries with many of the contributions (88%) coming from the US (58), Russia/Soviet Union (40), China (16), Japan (14), and Germany (13). The US was a major contributor in this research area (43% of reported findings) until the year 2000. Since that time, the US has contributed to only 27% of the reported findings. Several semiconductor materials (GaSb, GaAs, CdZnTe, InGaSb, etc.) have multiple listings as different crystallization conditions (method, time, temperature, etc.) were used for each listing. This current dataset underwent an initial, more detailed analysis. The authors acknowledge that the data presented here may be skewed toward a positive outcome bias^7^. Even with this caveat, a preliminary evaluation of the aggregated semiconductor data is performed here.

## Discussion

Improvements in crystals grown in microgravity versus terrestrially were evaluated using the following metrics: size, structurally better, more uniform, and improved performance (see Table 1). Of the 160 semiconductor materials fabricated in microgravity, 140 provide data in at least one metric. Given the diversity of applications, experimental goals, and discipline perspectives of the research compiled, not all reports provide data on the same metrics. Reports that did not include data on a particular metric were excluded. The number of crystals used for each evaluation are also reported. Several experiments (20) included none of the above metrics as they were primarily focused on the engineering process, crucible dewetting during crystallization, determination of appropriate magnetic field for preparing diluted magnetic semiconductors, and/or best temperature employed for crystal growth. Compounds that were reported as the “same” showed no change in crystal size, similar structures by optical measurements, and displayed similar performance to their ground grown counterparts. Leading examples for each of the characteristics are described below.

**Table 1:** Reported metrics for semiconductors

	Yes	No	Same
Larger (*n* = 46)	26 (57%)	18 (39%)	2 (4%)
More uniform (*n* = 89)	74 (83%)	15 (17%)	0
Structurally improved (*n* = 99)	84 (85%)	14 (14%)	1 (1%)
Improved performance (*n* = 41)	34 (83%)	3 (7%)	4 (10%)

Size is the metric where semiconductor growth in microgravity seems to show the least improvement. For a range of materials (Al-Bi-Sn, InSb-GaSb, CdZnTe, Ge-Si), the decrease in crystal size ranged from slightly smaller to a four-fold reduction^8^. Of the crystals that were larger in microgravity, the ability to maintain homogeneity with components of different densities in microgravity was often credited as the reason^9^.

When evaluating uniformity in semiconductor materials grown in microgravity verses terrestrially, several characteristics were reported. In several cases, physical characteristics such as the average apparent density/density profile were more consistent^10^, the crystallinity was comparable to “high quality crystals” of the same substrate^10^, and the size of inclusions were more consistent^11^. Other reports provided evidence that microgravity grown crystals were microscopically homogeneous^12^, there were more grain boundaries^11^, and voids were more uniformly distributed^13^. Examples of when dopants were added (Te and Ce), the distribution of the dopant was more consistent throughout the crystal than terrestrial analogs^11,14,15^.

Structural improvements were also reported for semiconductor materials. The observed improvements ranged from fewer impurity striations^14^, more homogeneous visually observable composition^11^, a lower etch pit density^15^, fewer macro-defects^14^, less twinning^13^, more regular faceting of the surface^16^, to a smoother surface^17^. In the dataset, there are examples of less dislocation density in microgravity samples^12,15,18^. Microgravity provided improved resistivity^19^, which may be related to fewer defects.

Improvements in the performance of the semiconductor materials were reported in over forty of the sources. As the applications for exotic semiconductors are numerous, there were a wide variety of evaluative measurements including electrical conductivity^20^, maximum mobility^20^, minimum carrier concentration^20^, resistivity^11^, photoconductivity^21^, photosensitivity^21^, transmittance^22^, and photoluminescence^23^. Specific applications into devices included radiation detectors^19^, double heterostructure lasers^12^, dual gate field effect transistors^12^, microwave integrated circuits^18^, and measurements of CO_2_ absorption^24^.

Given that researchers from crystallography, material science, physics, electrochemistry, and inorganic chemistry contribute to this field, a lack of consistency is observed in the reported metrics. For the purposes of this report, improvement was defined by each of the authors in the source material. The data used by the source authors to make these determinations ranged from microscopy to resistance measurements. This could mean that there are significant differences in what improvement means. Some experiments show improved results in one metric, while giving similar or decreased results in another^8^. Looking at improvement in one or more metric (Table 2), the benefits of microgravity grown crystals are substantial with more than 80% of experiments showing improvement. In addition, almost half of the microgravity experiments reported improvement in two metrics.

**Table 2:**
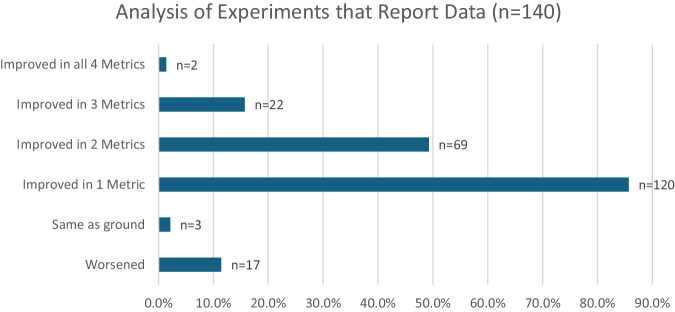
Overall improvements in metrics

Methods of producing semiconductor materials in microgravity were very diverse. Of the 160 literature reports, the vast majority (130) utilize melt methods (Bridgman, modified Bridgman, floating zone, traveling heater, etc.)^25^. A few (11) utilize vapor deposition or other vapor methods. Most of these approaches for semiconductor fabrication showed significant improvement in microgravity, regardless of method (Table 3). Only floating zone/zone melting methods showed slightly more than 50% improvement under microgravity conditions.

**Table 3:**
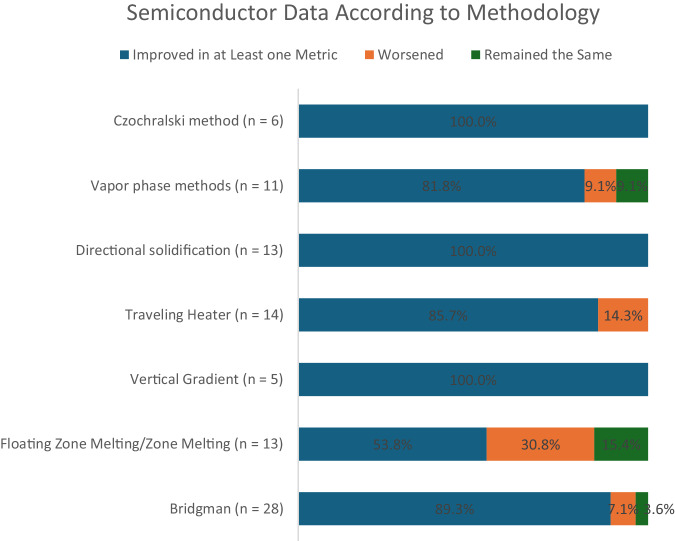
Improvements in metrics based on common methods utilized

Given the potential impact of improved performance, methods for producing semiconductors that are more uniform or improved structural quality are critical. The report of Chen, et al. stated “… these results have surpassed the best analog switch ICs [integrated circuits] made from terrestrially grown wafers in our laboratory … demonstrates the benefits of microgravity environment for growing semiconducting single crystals”^26^. Even accounting for a positive result bias in reporting, the data in Table 3 suggest that microgravity crystallization techniques offer significant benefits for semiconductor fabrication. By aggregating semiconductor data into a searchable, publicly available database, we provide a tool that allows for quick comparisons between a broad array of studies and applications.

## Data Availability

The datasets generated and analyzed during this study are available at the following repository: https://docs.google.com/spreadsheets/d/1Zl_B_IbC_UFx4VfFamhCQcGgUdtk64r6wOdQ_wO3lHU/edit#gid=218298295
